# A procedure for the detection of linkage with high density SNP arrays in a large pedigree with colorectal cancer

**DOI:** 10.1186/1471-2407-7-6

**Published:** 2007-01-12

**Authors:** Anneke Middeldorp, Shantie Jagmohan-Changur, Quinta Helmer, Heleen M van der Klift, Carli MJ Tops, Hans FA Vasen, Peter Devilee, Hans Morreau, Jeanine J Houwing-Duistermaat, Juul T Wijnen, Tom van Wezel

**Affiliations:** 1 Department of Pathology, Leiden University Medical Center, Leiden, The Netherlands; 2 Department of Human Genetics, Leiden University Medical Center, Leiden, The Netherlands; 3 Department of Medical Statistics and Bioinformatics, Leiden University Medical Center, Leiden, The Netherlands; 4 Department of Clinical Genetics, Leiden University Medical Center, Leiden, The Netherlands; 5 The Netherlands Foundation for the Detection of Hereditary Tumours, Leiden, The Netherlands

## Abstract

**Background:**

The apparent dominant model of colorectal cancer (CRC) inheritance in several large families, without mutations in known CRC susceptibility genes, suggests the presence of so far unidentified genes with strong or moderate effect on the development of CRC. Linkage analysis could lead to identification of susceptibility genes in such families. In comparison to classical linkage analysis with multi-allelic markers, single nucleotide polymorphism (SNP) arrays have increased information content and can be processed with higher throughput. Therefore, SNP arrays can be excellent tools for linkage analysis. However, the vast number of SNPs on the SNP arrays, combined with large informative pedigrees (e.g. >35–40 bits), presents us with a computational complexity that is challenging for existing statistical packages or even exceeds their capacity. We therefore setup a procedure for linkage analysis in large pedigrees and validated the method by genotyping using SNP arrays of a colorectal cancer family with a known *MLH1 *germ line mutation.

**Methods:**

Quality control of the genotype data was performed in Alohomora, Mega2 and SimWalk2, with removal of uninformative SNPs, Mendelian inconsistencies and Mendelian consistent errors, respectively. Linkage disequilibrium was measured by SNPLINK and Merlin. Parametric linkage analysis using two flanking markers was performed using MENDEL. For multipoint parametric linkage analysis and haplotype analysis, SimWalk2 was used.

**Results:**

On chromosome 3, in the *MLH1*-region, a LOD score of 1.9 was found by parametric linkage analysis using two flanking markers. On chromosome 11 a small region with LOD 1.1 was also detected. Upon linkage disequilibrium removal, multipoint linkage analysis yielded a LOD score of 2.1 in the *MLH1 *region, whereas the LOD score dropped to negative values in the region on chromosome 11. Subsequent haplotype analysis in the *MLH1 *region perfectly matched the mutation status of the family members.

**Conclusion:**

We developed a workflow for linkage analysis in large families using high-density SNP arrays and validated this workflow in a family with colorectal cancer. Linkage disequilibrium has to be removed when using SNP arrays, because it can falsely inflate the LOD score. Haplotype analysis is adequate and can predict the carrier status of the family members.

## Background

Colorectal cancer (CRC) is the one of the most common malignancies in the Western world. Already in 1913, familial aggregation of CRC was described by Warthin [1] and later Lynch *et al*. described an additional family with clustering of colorectal and endometrial cancer [2]. Clinical definition of Lynch syndrome, or HNPCC, in 1991 [3,4] was instrumental for linkage analysis, and ultimately for the identification of the underlying gene defects in HNPCC families. The first HNPCC loci were mapped to chromosomes 2 and 3 using microsatellite markers [5,6]. This eventually led to the identification of germ line mutations in *MSH2 *[7] and *MLH1 *[8], respectively. Later, *PMS2 *[9], *MSH6 *[10,11] and recently *MutYH *[12] were identified as CRC susceptibility genes. However, the so far identified CRC susceptibility genes can only explain up to 5% of all cases [13], while in ~35% of all colorectal cancer cases familial clustering is seen [14]. Furthermore, it is shown that first degree relatives of patients with colorectal cancer have a relative risk of 2.3 to develop the disease [15]. This indicates that still some genes with strong or moderate effect on CRC development remain to be identified. In order to identify these genes, linkage analysis in families could point to the loci where unknown susceptibility genes may reside. Indeed, different linkage analysis studies revealed potentially interesting regions on chromosomes 3q, 9q, 11q, 14q, 15q and 22q [16-20].

Families with a clustering of colorectal cancer but without germ line mutations in CRC genes have been under surveillance in Leiden since the 1980s. Due to the long period of follow-up, with three to four affected generations, these Dutch HNPCC-like families have become informative for linkage analysis.

Traditionally, linkage analysis is performed with multi-allelic microsatellite markers. Recently, however, the more advanced single nucleotide polymorphism (SNP) arrays were brought into use for linkage analysis. It was shown that the information content of a dense SNP map is significantly and uniformly higher than that of a genome wide microsatellite marker map [21]. Several studies conducting linkage analysis on genotype data from SNP arrays appeared in recent years [22-24]. In these studies non-parametric as well as parametric linkage analysis was performed in sib pairs or in small to moderate size pedigrees. However, to date, no studies have been published on linkage analysis using SNPs in large pedigrees (e.g. >35–40 bits).

Studying large families with thousands of SNPs results in a computational complex analysis that is challenging for existing statistical packages and that may even exceed their capacity. Current linkage analysis programs can handle either large pedigrees or large numbers of markers, depending on the underlying algorithm. In order to perform linkage analysis in large pedigrees using SNP arrays, we explored the possibilities of currently available linkage analysis software. Most currently available programs are based on the Lander-Green or the Elston-Stewart algorithm or both. The computation time of the former algorithm increases exponentially with the number of bits (2n - f, where 'n' is the number of non-founders and 'f' the number of founders) in a pedigree, whereas the latter scales exponentially with the number of markers. To perform multipoint linkage analysis in a large family with SNP arrays in one run would probably take several months computation time, if at all possible.

Several programs are suitable for linkage analysis with bi-allelic markers. Genehunter and Merlin can handle a relative large numbers of markers, however the analysis is restricted to pedigrees of up to ~30-bits [25,26]. Both programs are based on the Lander-Green Hidden Markov Model algorithm and can perform non-parametric as well as parametric linkage analysis. In Genehunter, the Elston-Stewart algorithm is also implemented, allowing the performance of simultaneous analysis of several markers as well as analysis of pedigrees of moderate size. A third program based on the Lander-Green Hidden Markov Model algorithm is Allegro 2. This program can handle large pedigrees (up to ~40 bits), although the computational costs increase substantially when not all genotype information of the family is available [27,28]. Allegro calculates parametric LOD scores as well as NPL scores and allele-sharing LOD scores. Another program, SNPLINK [28,29] can perform automated linkage analysis with LD removal using either Allegro or Merlin. However, for all the above mentioned programs the different branches of large families (i.e. >35–40 bits) need to be analyzed separately. This will lead to substantial loss of information and potential undetected linkage.

MENDEL [30] is a program that is suitable for linkage analysis with SNPs in large pedigrees. It allows adjusting the maximum number of meioses, though the computation time will increase in that case. Both parametric and non-parametric linkage analysis can be performed in MENDEL. The program will either use the Lander-Green or the Elston-Stewart algorithm, depending on whichever is more efficient for the pedigree. SimWalk2 is a program that can perform multipoint parametric linkage analysis, haplotype analysis and a few other analyses in large pedigrees using bi-allelic markers. It uses Markov chain Monte Carlo methods to compute the likelihood [31]. Simwalk2 uses the MENDEL program for computing location scores. With the aim to detect linkage in CRC families exceeding 40 bits we established a procedure using freely available software packages and validated this in a large colorectal cancer family, with a known causal *MLH1 *germ line mutation on chromosome 3.

## Methods

### Patients

**Figure 1:**
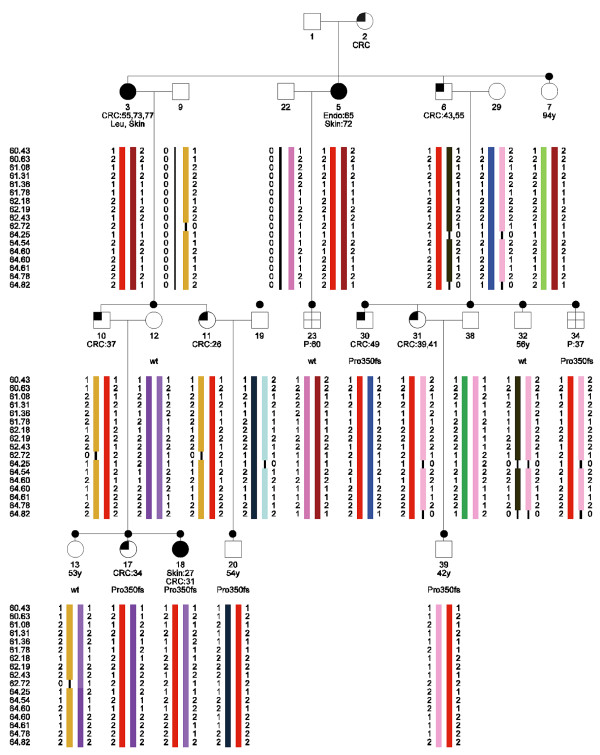
**Haplotype analysis in a HNPCC family segregating the *MLH1 *Pro350fs mutation**. The haplotypes were constructed in SimWalk2 and subsequently visualized with HaploPainter [39]. CRC:55, colorectal cancer diagnosed at age 55; Endo, endometrial cancer; Skin, skin cancer; P, polyps; Pro350fs, carrier of the Pro350fs mutation in *MLH1*; wt, non-carrier; black dot, DNA of this family member has been typed on a 10K SNP array.

A large colorectal cancer family (Figure 1) with a recently identified mutation in the *MLH1 *gene (c.1046dupT, p.Pro350fs) was studied. Nine family members are affected with colorectal cancer. Another two family members are affected with polyps and three cases with skin cancer (non-specified) and one case with endometrium cancer (non-specified) are seen as well. Peripheral blood lymphocytes were collected from the family members. DNA was extracted using standard procedures. A total of thirteen family members were genotyped on Affymetrix GeneChip Human Mapping 10K 2.0 SNP arrays. The arrays were processed according to the instructions of the manufacturer. The mean SNP call rate was 96.3% (89.0%-98.5%).

The study was approved by the Medical Ethical Committee of the LUMC (protocol P01-019).

### Workflow

We processed the data according to the following workflow: 1) First, the genotype data were generated by GeneChip DNA Analysis Software (GDAS) from Affymetrix. 2) These genotype data were combined with the pedigree and the marker information in Alohomora. 3) In this program the uninformative SNPs were removed as well. 4) To be able to perform linkage analysis in the desired program, the output files (in Merlin-format) of Alohomora were by Mega2 converted to the proper format. 5) Mega2 also removed the Mendelian inconsistent errors. 6) The files were then ready to perform parametric linkage analysis using 2 flanking markers in MENDEL; affected-only analysis as well as parametric linkage analysis using liability classes was performed. 7) Based on the second analysis, regions of interest were defined that were further tested for Mendelian consistent errors and 8) possible linkage disequilibrium was removed in SNPLINK. 9) Multipoint parametric linkage analysis using the liability classes was then performed in Simwalk2 for the ROIs and 10) finally, the haplotypes were inferred in Simwalk2.

### Data formatting and quality control

Genotype data of the individual family members were generated using GeneChip DNA Analysis Software (GDAS) from Affymetrix. In the Alohomora program [32] the pedigree information, allele frequencies and map position of the SNPs were combined with the genotype data generated by GDAS. The uninformative SNPs in this pedigree, that show either only A alleles and No Calls or only B alleles and No Calls, were removed from further analysis by Alohomora. The data files were exported in Merlin format. Subsequently, in Mega2 [33] these Alohomora files were converted into the appropriate format for the programs used for linkage analysis, i.e. either the Mendel 5 format or the SimWalk2 format. Mendelian inconsistent errors were removed from analysis with Mega2 by setting all genotypes of these SNPs to unknown.

### Mendelian consistent errors

Mendelian consistent errors were identified by mistyping analysis. Since this analysis is computationally complex and therefore time consuming (2 1/4 hours for 35 SNPs), only the regions of interest were analyzed for Mendelian consistent errors. All chromosomal regions with LOD scores exceeding 1 and lacking negative LOD scores were defined as regions of interest (ROI). SimWalk2 [31] was used to check all ROI for Mendelian consistent errors by performing mistyping analysis. An error model with a uniform error rate for all mistypings was used. The overall rate of mistyping was set at 0.004 [34,35]. The threshold for the posterior probability of mistyping was set at 0.5 [36].

### Linkage disequilibrium estimation

In the ROI the pair-wise correlation coefficient r^2^, as a measure of linkage disequilibrium (LD) between adjacent SNPs, was estimated using SNPLINK [29] and Merlin [26]. Since we are only interested in estimates of r^2^, we split the large family into nuclear families. In addition to the family under study, genotypes from 12 Dutch nuclear families from other studies (unpublished results) were used to calculate LD. The program SNPLINK provides a list of SNPs to be removed. We used as cut off value for LD removal an r^2 ^≥ 0.4. The information content was computed before and after removal of the SNPs using Merlin.

### Linkage analysis

To determine the power to detect linkage in the *MLH1 *family, we performed a simulation study using Simlink [37] under the assumption of a dominant trait with a piecewise linear penetrance. Subsequently, we performed an affected-only linkage analysis and modeled a dominant trait with an allele frequency of 0.001. For parametric linkage analysis, the proper assignment of affected status to family members is crucial since, due to the surveillance of the families, adenomas will be detected and removed before they can develop into a carcinoma. Additionally, the risk of cancer increases with age. And the risk of developing an adenoma is different from the risk of developing a carcinoma. To adjust for these phenomena, we defined 10 liability classes: four classes were defined with different penetrances for colorectal cancer; four classes for polyp carriers and two more liability classes for spouses, that carry a population risk of developing polyps or colorectal cancer and one for the family members of which the disease status is not known. These liability classes are based on the incidences of CRC and adenomas in the members of HNPCC families in the Netherlands, that do not carry the disease causing mutation [38].

In MENDEL [30], an affected-only parametric linkage analysis was performed using two flanking markers (computation time: ~20 sec per chromosome). In this analysis only family members with colorectal cancer were defined as affected and all other persons were set to unknown. Parametric linkage analysis with liability classes was performed thereafter, using two flanking markers (computation time: ~20 sec per chromosome). Cancers other than colorectal cancer were not considered to be part of the syndrome. In the ROIs appearing from this linkage analysis, possible Mendelian consistent errors were removed as well as the possible presence of linkage disequilibrium. Subsequently, multipoint parametric linkage analysis was performed in SimWalk2 [31], using the ten liability classes. In this multipoint analysis no more than 30 SNPs were analyzed, limited by the computational complexity (analysis time: 1 3/4 hours for 30 SNPs).

### Haplotype analysis

Haplotype analysis was performed in the ROI, using SimWalk2. All SNPs in the region of interest (~18) were included in this analysis (computation time: 1 1/3 hours for 18 SNPs). The results of the haplotyping were visualized in HaploPainter [39]. The haplotype segregation in the family could then be compared to the segregation of the mutation in *MLH1 *in this family.

## Results and discussion

Linkage analysis using bi-allelic genotype data from SNP arrays and large families is a computational challenge using commonly used, freely available analysis software. For the different steps of the linkage analysis; e.g. data formatting, detection of Mendelian inconsistencies, mistyping analysis, LD removal and single to multipoint linkage analysis, we have chosen the following programs that can handle large pedigrees and many SNPs where required; Alohomora [32], Mega2 [33], MENDEL [30], SNPLINK [29] and SimWalk2 [31].

In advance of the linkage analysis we performed a simulation study to calculate the power using Simlink. The mean LOD score in 1000 simulations in this family was 2.0.

The Alohomora program [32] was used first to combine the genotype data generated with the SNP arrays, and the pedigree and SNP information and secondly, to convert these data into the appropriate format for further analysis. In addition, 1256 of the 10053 SNPs were uninformative and were therefore removed from analysis by Alohomora.

Since errors in genotyping can easily mask linkage, the data were checked for different types of errors. First, we have estimated the genotyping error rate in five duplicate experiments. The mean genotyping error rate between the duplicates was only 0.0051.

Mega2 was then used for several data validation checks, including errors in the pedigree data or Mendelian inconsistent errors. Mega2 was used since it supports 28 different programs, including the programs MENDEL and SimWalk2, which we have used for linkage analysis and haplotype analysis. The genotypes of 18 SNPs (0.21%) were removed from analysis, because of Mendelian inconsistencies. However, with bi-allelic markers not all errors appear as Mendelian inconsistent errors [40]. The data were therefore also checked for Mendelian consistent errors. Because of the computational complexity of these multipoint analyses, this error check was performed only in the regions of interest. The mistyping analysis option in SimWalk2 was used, since this program can handle such a complex analysis in a large pedigree. No Mendelian consistent errors were identified in the ROI.

**Figure 2:**
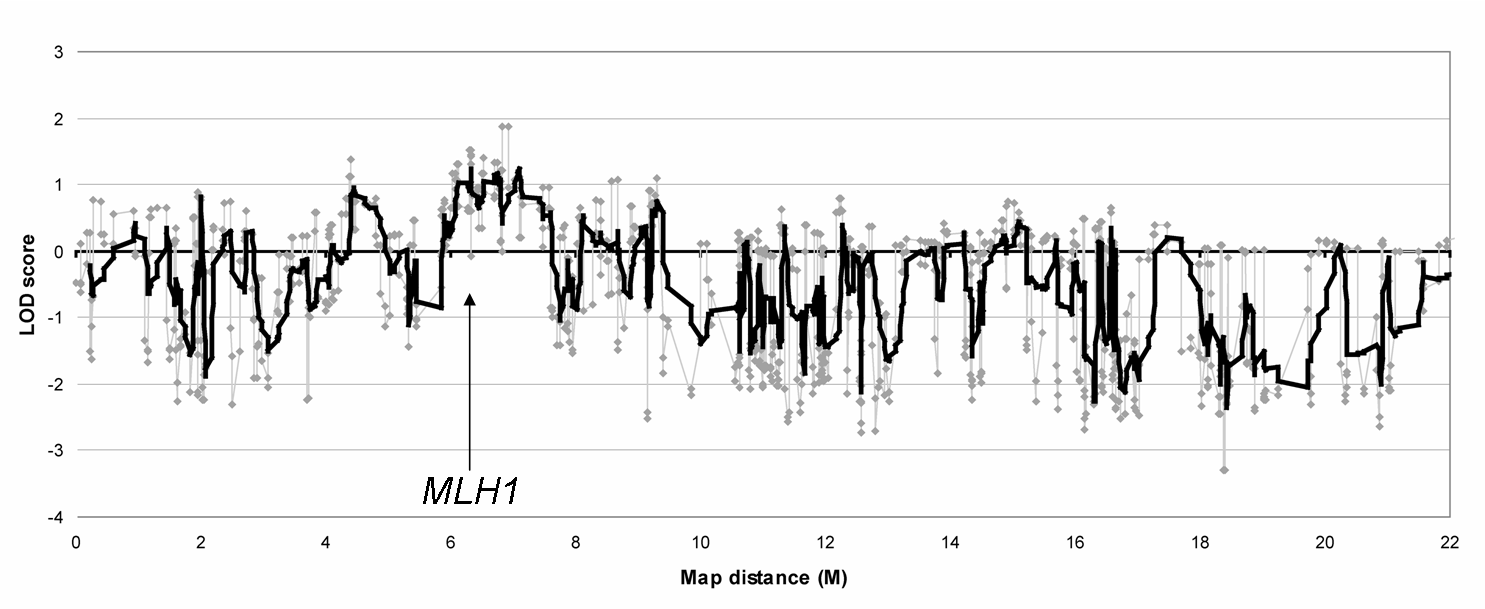
**Parametric linkage analysis on chromosome 3, using two flanking markers**. The maximum LOD score is 1.9. The gray line represents the raw results of the linkage analysis. The black line is the moving average with a period of ten.

Affected-only parametric linkage analysis and parametric linkage analysis using liability classes was performed in MENDEL, using two flanking markers. This analysis showed a maximum LOD score of 1.8 in the affected-only analysis and 1.9 using liability classes for a 1.7 Mb region around the *MLH1 *gene on chromosome 3 (Figure 2). A second region with a LOD 1.1 was found, both in the affected-only analysis and using liability classes, near the centromere on chromosome 11.

Current linkage analysis programs assume LD between markers and a disease locus and importantly, linkage equilibrium between markers. The presence of linkage disequilibrium between two markers can falsely inflate the LOD score and missing genotypes can increase this effect. Therefore, the r^2 ^as a measure of LD was computed in Merlin and SNPLINK. Using the threshold r^2 ^≥ 0.4, 5 of the 27 SNPs in the region on chromosome 3 were removed from the analysis. From the region of interest on chromosome 11, 14 of the 30 SNPs with an r^2 ^≥ 0.4 were removed from the analysis. After LD removal, multipoint linkage analysis in the region on chromosome 3 yielded a LOD score of 2.1, whereas on chromosome 11 negative LOD scores were seen by multipoint linkage analysis after LD was removed. This indicates that the strong LD in the region on chromosome 11 was responsible for the peak in the LOD in that region. On both chromosomes, the removal of SNPs with high LD had no significant effect on the information content (not shown).

We inferred the haplotypes of the family members, using SimWalk2 for the linkage region on chromosome 3. All known affected *MLH1*-mutation carriers share the same haplotype, as well as the affected obligate carriers. Therefore, this haplotype perfectly co-segregates with the clinical phenotype of the family members (Figure 1). Case 23, who had developed polyps at age 60, does not share this haplotype. Subsequent mutation analysis showed that this individual indeed did not carry the disease causing mutation in *MLH1*. Therefore, this case showed to be a phenocopy. Another family member, case 39, has to date not developed clinical symptoms of HNPCC, although he did inherit the disease causing allele according to the haplotype analysis. Indeed, sequence analysis showed that this person carries the mutation.

## Conclusion

In conclusion, we show that we can perform linkage analysis with high-density 10K SNP arrays in large families for which not all members could be genotyped. We developed a workflow with different publicly available software to perform the analyses: removal of Mendelian consistent and Mendelian inconsistent errors, two and multipoint parametric linkage analysis, removal of linkage disequilibrium and haplotype analysis. The procedure was validated in a large CRC family carrying a known germ line mutation in *MLH1*. Linkage was found with the *MLH1 *gene and subsequent haplotype analysis corresponds to the mutation status of the family members. This procedure can now be used for linkage analysis of large families with an inherited condition, such as hereditary colorectal cancer.

## List of abbreviations

CRC; colorectal cancer

SNP; single nucleotide polymorphism

LD; linkage disequilibrium

LOD; log of odds

HNPCC; hereditary nonpolyposis colorectal cancer

GDAS; GeneChip DNA Analysis Software

ROI; regions of interest

## Competing interests

The author(s) declare that they have no competing interests.

## Authors' contributions

AM performed SNP arrays and mutation analysis, statistical analyses and drafted the manuscript. SJC and QH assisted in the statistical analysis, and QH performed the LD analysis. HMVDK performed SNP arrays. CMJT provided DNA samples and mutation status. HFAV was responsible for family recruitment and surveillance. PD participated in study design. JJHD supervised the statistical analysis, JTW, HM and TVW designed and coordinated the study, TVW helped to draft the manuscript. All authors read and approved the final manuscript.

## Pre-publication history

The pre-publication history for this paper can be accessed here:
